# Coping with drought: Reflection of communal cattle farmers in Umzingwane district in Zimbabwe

**DOI:** 10.4102/jamba.v11i1.813

**Published:** 2019-10-15

**Authors:** Thabo Ndlovu

**Affiliations:** 1 Institute of Development Studies, National University of Science and Technology, Bulawayo, Zimbabwe

**Keywords:** capitals, coping, drought, resilience, vulnerability

## Abstract

The frequency of drought presents huge challenges to rural farming communities in rural Africa. To circumvent negatives of drought, rural Zimbabwean farmers have devised coping strategies some of which are detrimental to the cattle enterprise. Using the sustainable livelihoods framework, this article sought to examine prevalent coping strategies in Umzingwane district in Zimbabwe, some of which lay a foundation for building resilience drought. Qualitative approaches underpinned by descriptive survey design guided data collection processes with structured and non-structured questionnaires administered to unravel drought coping behaviours of rural farmers. The findings reflect that farmers largely rely on moisture-sensitive coping strategies, an indication that rural farmers have not embraced contemporary cattle management practices. The coping strategies reflect a poorly engaged farming community which has been relegated to mere spectators in the industry, despite owning a significant fraction of the national herd. Communal farmers are encouraged to embrace savings clubs, insurance and fodder banks concepts to complement natural grazing and decisively deal with drought by spreading the risks and investing in proactive processes.

## Introduction

Drought, the world over, is one of the most slow-onset and devastating phenomena characterised by extreme moisture deficits that normally disrupt rural livelihoods. Rural farmers in Africa and beyond experience a plethora of drought impacts, making coping an inescapable discourse. Yilma et al. (2014) view coping as actions undertaken by households to accommodate the effects of a shock, while Dercon (2002), Eriksen, Brown and Kelly (2005) and Pelling (2011) deem it as a short-term survival strategy engaged within the existing structural context in response to extremes. Park (2011) concurs that coping prevails when an individual redefines an incident so that it fits within their survival matrix. Coping signifies the rooms to manoeuvre, ability to absorb, accommodate and recover from drought impacts (Smith et al. 2000; Thomas & Twyman 2005). Coping can be triggered by stress factors or it may be instituted to strengthen already existing inbuilt approaches. Ellis (1998) considers coping as impromptu reactions to rapid shocks, while Dercon (2002:145) avers that ‘it’s success in dealing with a crisis’, as it compels the affected to adjust to meet physiological, social, economic and political needs of drought (Masendeke & Shoko 2013a). This article sought to unravel drought coping strategies practiced preferred by communal farmers in Umzingwane district and their contribution in strengthening foundations to build resilience.

Coping strategies are categorised as problem- and emotion-based. Problem-based coping strategies demand taking actions to transform the shock creating the distress, whereas emotion-based coping concerns governing distressing emotions (Becker, Roos & Coetzee 2015). Avoidant coping involves isolating oneself psychologically from the stressor by paying attention to something else other than the source of the stress (Scott et al. 2010). Zarafshani, Gorgievski and Zamani (2007) suggest that communal farmers adopt emotion-focused strategies because they view drought as uncontrollable and something that can be endured. Such attitudes largely result in farmers losing more assets as they resort to acceptance and praying for rain, and this is a common practice in rural Zimbabwe. Not only do they pray, traditional ceremonies are part of the package to appease ancestral spirits in anticipation of adequate to above normal precipitation. Appraising a hazard as a threat contributes to less emotion-inclined coping as it normally foster protective functions through adoption of high-risk coping measures (Bjorck & Klewicki 1997). This coping mechanism opens a way for extension agents to impart knowledge and proffer diverse survival skills (Zarafshani et al. 2007).

Coelho, Adair and Mocellin (2004) attest that communities in drought-prone areas have lower levels of threat perception than those living in less drought risky areas. Zarafshani et al. (2007:11) corroborate that ‘high drought intensity farmers seek less social support and advise from professional bodies’ an indication that they require special skill and capitals to deal with hazards. The article used the sustainable livelihoods framework (SLF) to anchor discussions on drought coping initiatives in communal farming areas. The SLF as indicated by Scoones (1998) is widely applied when analysing the nexus between livelihoods and resource use. The framework helps to initiate livelihood’s buffer measures against environmental, social and economic stresses (Morse & McNamara 2013). Coping mechanisms are intricately linked with the local resource base; hence, the SLF proved very useful to untangle access, endowment and deployment of resources to build drought resilience (Woodhouse et al. 2000). In addition, the SLF groups assets into five capitals, namely natural, physical, human, financial and social (FAO 2008). Capitals are viewed as inputs and/or outcomes for livelihoods (Morse et al. 2009). The framework’s relevance was drawn from the notion that coping with drought is a reflection of assets endowment, their deployment that influences resilience strengthening in rural livestock farming. In this regard, the SLF facilitated discernment of resources and means which cattle farmers use to mitigate stressors (FAO 2002) and assets through which rural livelihoods are sustained to enhance drought resilience (Biggs et al. 2014).

Umzingwane is a drought-prone district, receiving less than 450 mm of rainfall annually with livestock farming a dominant source of livelihood. The area is rocky, characterised by gully erosion with some parts of the district severely affected by deforestation, exposing the land to erosion elements. There is diversity on the means of survival in the area with dry land cropping, livestock farming, artisanal mining, fishing and vending the dominant activities. The harvesting of natural resources such as veld and gold deposits is conducted unsustainably resulting in increased siltation of major water supply dams and rivers. The water-holding capacity has been reduced drastically by artisanal mining deposits and indiscriminate cutting down of trees and overgrazing as evidenced by animals walking long distances in search of veld and at times straying into neighbouring areas as they battle with drought shocks.

## Coping and drought resilience link

Resilience, whose genesis is the Latin root resi-lire, implies to spring back (Davoudi et al. 2012). Like vulnerability, resilience has no generally agreed definition; however, its conceptualisation varies with disciplines. Resilience processes embrace copings strategies which lay a sound foundation for transformation of vulnerable communities through learning and ultimately adaptation. Holling (1973) defines resilience as a measure of the persistence of systems and of their ability to absorb change and disturbance and still maintain the same level of function. It is essential to note that in some fields such as engineering, resilience is narrowly used to mean return to equilibrium, while some construe it as bouncing back better after a shock (Folke 2016). In a business setup in which communal farmers are unwittingly part of, resilience is conceived as the capacity of a unit to survive, adapt and grow in turbulent circumstances (Fiksel 2006). There is an expectation for coping processes to galvanise pliability in dealing with perturbations and bolster-adaptive capacity of communal cattle farmers to sustainably dissipate shocks through institutions and networks that learn and store knowledge and experience (Resilience Alliance 2014). The appropriateness and relevance of coping interventions is reflected by the impact of the hazard and informs on competitive buffer mechanisms. Central to the discussion is paying attention to the view that coping should not reduce drought risks of the cattle enterprise at the same time cause it to be less resilient in other ways (Cifdaloz et al. 2010). In this study, coping actions such as the use of crop residues, destocking, savings, credit lines, transhumance, supplementary feeding, shared grazing and fodder banks were interrogated to discern their influence on drought adaptation.

## Drought coping practices in communal areas

Drought challenges in rural Africa date back to time immemorial, with Zimbabwe escalating extension services provision post-independence to arrest the scourge. Part of the means to contain drought involved destocking, which Catley and Cullis (2012) define as the intentional removal of animals from the range in times of distress before they succumb and become worthless. Shutt (2002) corroborates that by the mid-1930s, the perception of environmental dilapidation grew and this accelerated momentum of destocking campaigns. Culling of cattle severely interfered with the Ndebele tribe’s (dominant in Umzingwane) social economy which is largely anchored on livestock as a symbol of wealth and social status (Mapuva 2015). The discussion makes reference to a significant publication in 1926 by Melvin Herskovits that farmers in East Africa displayed a strong cultural fondness for their herds which challenged economic rationality (Shutt 2002). In an effort to enforce coping through destocking, the Rhodesian government enacted the *Land Husbandry Act* (LHA) of 1951 (Selby 2006) which provided for the establishment of conservation measures in communal areas. Utete (2003) and Luthe (2007) concur that the *LHA* of 1951 was meant to impose construction of conservation works and control stock numbers in communal areas. The *LHA* was not well conceived by communal residents and it triggered much anger which culminated in the negativity on destocking being witnessed today in rural Zimbabwe (Luthe 2007). Consequently, despite that most colonial legislation has been set aside, they still haunt rural farmers and largely impact on their coping behaviours and attitude towards certain livestock management practices (Mapuva 2015). Postcolonial land management legislative frameworks promote management of herds by households within administrative boundaries of local villages, wards and districts (Mabhena 2014). Apparently, the land redistribution programme post-year 2000 has added other complexities regarding cattle management with arguments that it has not addressed cattle congestion making destocking a feasible alternative when mitigating drought.

The disposal of assets to contain drought shocks remains a contested decision as it becomes a common buffer in the short run, though breeding long-term dire consequences. Masendeke and Shoko (2013) opine that small livestock like chickens are the first to be disposed of, with bigger possessions such as cattle and agriculture implements sold later as the situation deteriorates. While destocking helps reduce the need for more grazing land, thus plummeting pressure on veld, farmers in communal circles find it absurd to sell cattle largely because of exorbitant restocking costs, small herd sizes, diminishing draft power availability and limited knowledge on the length of drought periods (Moyo, Dube & Moyo 2013). While destocking has been rarely implemented, Moyo et al. (2013) and Campbell et al. (2000) suggest that it is a common process amongst researchers and policy makers in government because of their insensitivity to the prime objective of livestock ownership.

Switching to hardier breeds is less common as a coping and adaptation measure, because of the high cost of replacement and non-availability of preferred breeds in the vicinity (Clarke, Shackleton & Powell 2012). This is in contrast to suggestions by O’Farrell et al. (2009) that switching to hardier breeds (Afrikaner & Nguni), diversifying economic activities and renting additional land are common drought coping and adaptation strategies. For communal farmers, investing in indigenous breeds is noble as these are tolerant to harsh conditions, though they are said to carry less weight which generates a smaller amount of income on the market (Clarke et al. 2012).

Transhumance is an opportunistic management practice that involves moving with animals to valleys of major rivers during winter with herders creating temporary shelter when tending their animals during periods of low graze (Moyo et al. 2013). The practice has become less popular in Umzingwane as most of the land was parcelled out during the 2000/2001 Fast Tract Land redistribution exercise. Grazing has depreciated in most communal areas resulting in livestock value depreciating and some breeds becoming extinct (IRIN 2013). Transhumance was common in yester years when land was enough to accommodate the practice without generating inter- and intra-community conflicts and entrenching vulnerability.

Vulnerability, a condition that predisposes cattle farmers to drought risks, highlights susceptibility of the cattle enterprise to the rise in frequency and intensity of drought in the area (Omondi et al. 2014). To decimate vulnerability, accessing credit lines from financial institutions is encouraged as it defers the adverse results of drought. Despite this benefit, Yilma et al. (2014) suggest that most households in communal areas consider borrowing as a last resort. The fear generated by the absence of collateral in communal farming areas and short loan repayment periods contributes to the few numbers of borrowers. The establishment of the Agriculture Bank of Zimbabwe (ABZ) in 1996 in terms of the *Banking Act of Zimbabwe* (Chapter 24:20) was one of the drought amelioration strategies meant to support and cushion commercial and communal farmers through enhanced access to credit. The ABZ has in times of drought-provided restocking finances for communal farmers at affordable rates. The challenge with the initiative is that of the limited number of applicants considered against those in dire need of financial support to sustain drought mitigation costs.

Supplementary feeding is most popular with commercial farmers in Umzingwane. Lusby (1990) describes supplementation as an activity of buying commercial livestock feed to cover the deficit ensuing from poor vegetative growth in natural grazing. For financially constrained farmers, this practice lasts for a short period. Horn, Hart and Paisley (2003) dispute the objective of supplementary feeding, arguing that it hinders destocking and ultimately contributes to over-stocking and land degradation. The high costs of supplementary feeding force some communal farmers to rely on gifts. Gifts from fellow communal farmers, friends, formal and informal groups are a rare livestock drought coping activity with studies, suggesting its dependence to be as low as 2% though it may rise depending on the shock, for example, health may be 5% (De Weerdt & Dercon 2006; World Bank 2013). Relying on gifts is a challenge especially, when a shock affects the entire community (Yilma et al. 2014).

## Methodology

In interrogating coping mechanisms common in communal cattle farming areas, the descriptive research design was adopted and qualitative data collection processes were used. The data gathering processes widened space for communal farmers to express their priorities and perceptions on drought coping practices. The descriptive survey which ‘can either be qualitative or quantitative’ (Wilson 2014:118) offered flexibility on the choice of data collection instruments during which structured and non-structured questionnaires were administered. The descriptive research design was relevant as it facilitated the use of stratified random and purposive sampling. The study area and key informants were purposively targeted for in-depth and focus group interview processes to generate insights on farmer coping patterns. Through purposive sampling, representatives from Umzingwane district, particularly Sigola, Sibomvu, Mawabeni and Silobi wards, together with information-rich respondents from institutions such as Department of Crop and Livestock Services (AGRITEX), Environmental Management Agency (EMA), local chiefs and village heads, were identified to inform on livestock practices and their contribution to building resilience to drought. Selection was based on cattle ownership, grazing conditions and decision-making roles, one’s experience and contribution to livestock management. The method aided the strategic selection of information-rich cases which illuminated issues being investigated (Patton 2015).

For structured questionnaires, a sample size of 180 communal cattle owners was drawn from six dip tanks with a total population of 3324 stock owners, translating to an average of 30 respondents per dip tank. Livestock owners in Umzingwane communal areas are easily identifiable in relation to where they dip their livestock, and hence this informed the sampling process. Strydom and Venter (2002:200–201) aver that ‘the size of the sample needs to be 450 of the population of 10,000 to be representative’. Guided by this statement, a 5% sample size was targeted using a stratified random technique to generate meaningful conclusions. Research participants were divided according to gender and years of experience in livestock management. Each stratum was subjected to simple random sampling to offer every element a fair chance of participating in the study. The veterinary cattle list was used and every communal cattle owner was assigned a number that was put into a hat from which participants were randomly picked until the target was satisfied. Stratified random sampling facilitated the involvement of female farmers to discern their perceptions on drought coping mechanisms employed by the communities. Precision on variables under study was enhanced by the use of stratified randomly selected respondents for whom structured questionnaires were administered. To buttress structured interviews, six focus group discussions and 20 in-depth interviews targeting those knowledgeable and involved in cattle farming as technical advisors, policy makers, representatives of formal and informal livestock management institutions were conducted as complements. Open-ended responses were analysed through grouping into themes and applying the process of eliminating irrelevant responses. The analysis experienced unexpected and hard to classify themes which were interconnected into narratives that explained coping of communal farmers with livestock drought. Structured data were processed with the aid of IBM SPSS software. The IBM SPSS software facilitated the generation of figures which detailed more insights on coping strategies embraced by the community. Community-preferred drought coping interventions were explained together with what they know but rarely apply when faced with drought situations.

### Ethical considerations

The author sought clearance from the Zimbabwe provincial and district structures as well as the University of the Free State. The responses to the questionnaire remain confidential and were aggregated in the final analysis. The respondent’s consent was sought. Furthermore, respondents were free to skip questions they were not comfortable answering and were at liberty to discontinue the interview at any time. It took a maximum of 45 minutes to administer the questionnaires and in-depth interviews.

## Results and discussion

### Drought coping dynamics in communal farming areas

**FIGURE 1:**
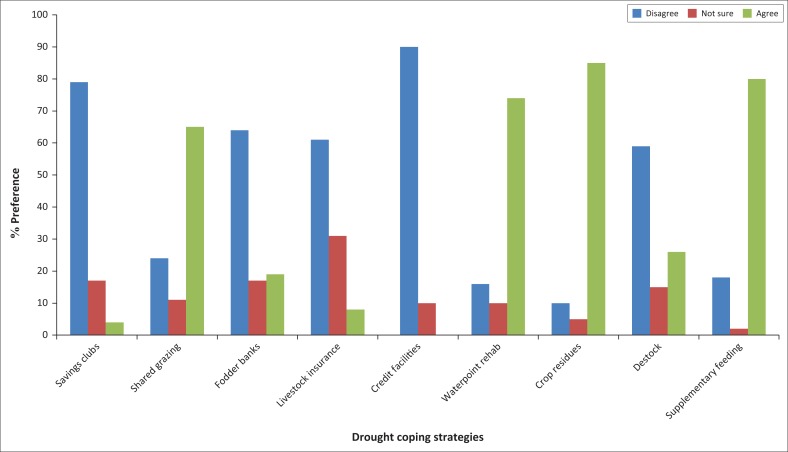
Cattle drought coping strategies.

Communal farmers react differently to drought situations. To cope with drought impacts, communal farmers are compelled to make choices of which amongst them affordability and synchrony of proposed solutions influences the decision. The advent of climate change is deepening drought vulnerability at the same time setting the tone for coping and adoption of strategies that foster resilience strengthening to proffer the cattle sector competitive advantage should drought strike. Figure 1 highlights coping strategies implemented by communal farmers when faced with perturbations.

Communal resource endowment, deployment and limitations are reflected by farmer choices efforts to contain drought. The use of crop residues, supplementary feeding, water point rehabilitation and shared grazing were opted because of factors of endogenous and exogenous nature to farmers. Savings clubs, fodder bank establishment and cattle destocking are known but rarely exercised by local farmers as detailed below.

### Crop residues

Gathering and preservation of crop residues is a common short-term winter-feed measure embraced by the majority of communal cattle farmers. Crop residues availability is constrained by the frequency of droughts subsequently drowning chances of having enough to sustain cattle throughout the dry season. Farmers averred: ‘what can we do, inputs are received late and the yield is not enough’ (Farmer, female, December 2017). Communal farmers have witnessed late planting coupled with poor animal management practices as livestock stray and contribute to low stover in winter. This practice is not necessarily a drought mitigation factor but is common as a winter feed. In support, Valbuena et al. (2012) and Masikati (2011) hint that crop residues are a prevalent source for winter feeding in communal areas and very pronounced cattle feed in sub-tropical Africa and Asia.

Despite crop residues being a common drought coping strategy, its sensitivity to moisture variation makes it highly undependable. Reliance on the practice signifies farmer’s limited knowledge and options as it further entrenches vulnerability of cattle to drought in communal areas. To augment crop residues, communal farmers acknowledged that cutting of green grass to make hay was another option they were aware of despite that it is rarely practiced because of poor supportive resources. Apart from farmer’s limitations, transforming structures ought to provide technical support to enhance the nutritional value of crop residues and help devise means to ensure that they last longer.

### Supplementary feeding

Supplementary feeding, a popular drought coping measure, favoured by those with sound financial resources as they grabbed as much as they could ahead of the incapacitated who had access but could not afford. The persistency of drought has rendered supplementary feeding not a viable strategy for communal farmers as it increases the duration and costs of supplementation. One of the traditional leaders said ‘the majority have no income to buy stock feed’. (Traditional leader, male, December 2017). This implies that farmers lack financial capital to pursue different options of saving animals against drought with supplementary feeding included. Timing and means of providing supplementary feeding need remodelling to circumvent the generation and perpetuation of vulnerable situations in communal areas. Supplementary resources mainly subsidised by government, private institutions and Non-Governmental Organisations (NGO) ought to be evidence-based to inform timing and targeting of vulnerable situations to effectively cushion livestock against drought. Assistance should categorise farmers based on level of least to most vulnerable to design supplementary programmes that suit the farmers’ circumstances. Wilhite, Sivakumar and Pulwarty (2014) concur that emergency resources be administered in ways that are anchored on the principles of drought risk reduction, as less attention has been paid to resilience building. Short-term coping strategies like supplements provision should embrace the resilience ideology to minimise generating dependency to external resources. This can be achieved through prioritising supplements for breeding stock at the same time encouraging farmers to dispose non-productive herds. Productive herds are critical to support post-drought recovery initiatives; hence, community participation becomes inescapable in ever-changing farmers’ circumstances and evolving coping strategies. Amare, Mekuria and Belay (2017) profess that greater interrogation and understanding of communal farmers’ insights and attitudes on drought response is vital to invent compatible coping means.

### Water reticulation

The rehabilitation of water points is one of the coping means adopted by communal cattle farmers in Umzingwane. The limited access to safe drinking water for cattle compels communities to repair boreholes and sink wells along rivers dotted around the district minimise shortages of the precious liquid. The presence of human capital in the form of locally trained pump minders provides the expertise to rehabilitate water points with minimal assistance from quasi-government institutions such as the District Development Fund and other development agencies. In an effort to spread and improve usage of grazing resources, rehabilitation of water points was crippled by the shortage of spares and equipment to address water accessibility constraints. The scarcity of water during dry period’s increases livestock mobility and it tends to concentrate grazing close to water sources. The competition for water with other sectors especially artisanal miners compounds the water crisis in this area in drought situations.

Masikati (2011) concurs that water limitation becomes widespread during the dry periods forcing animals to spread and travel approximately 14 km per day in search of water. Not only is the situation compounded by drying of water sources, it is the poorly developed water infrastructure that increases competition for the precious liquid, triggering repeated use of certain grazing resources and exposing animals to diseases. Morokong (2016) is of the view that well-developed cattle water systems diversify feeding sources which, in turn, cuts on the distances travelled in search of water and feed. By virtue of being located on dry land, exposure to drought is very high hence adaptation mechanisms to proactively reduce susceptibility to water and feed shortages is paramount through investing in water harvesting technologies.

### Shared grazing

Sharing of grazing resources practiced in Umzingwane as a livestock survival strategy allows farmers to drive vulnerable cattle to far better feeding areas. In-depth discussions confirmed that while the practice is common, it has the potential to create inter-community conflicts because of increased competition for grazing. Some neighbouring farms allow communal farmers to rent grazing space for a defined period. Respondents were mum on the amount charged per graze per animal. The migration of cattle in search of forage is not unique to this area alone as confirmed by Moyo et al. (2013) that the movement of livestock in search of better grass is common in most parts of Matabeleland South communal areas because of poor livestock infrastructure. The lack of fences to demarcate grazing boundaries works in favour of those with limited grazing space as a high number of their stock stray into areas reserved for future use by settlers in neighbouring areas. The challenge with shared grazing is that livestock contracts and spreads diseases as they invade new territories. Failure to discern by-laws has eroded the idea of sharing as outsiders are mostly accused of grazing livestock beyond the stipulated durations and zones. However, it remains a common strategy of ameliorating drought despite the conflicts it generates.

### Destocking

Destocking is not the preferred route to buffer cattle against drought. Interviews with locals aver that cattle destocking was not popular as most farmers do not have enough numbers to dispose such that those that managed to sell, they did so to deal with pressing issues such as school fees and other social problems needing urgent attention. Destocking relieves memories of the colonial laws such as the *LHA* of 1951 which restricted households from keeping a defined number of livestock. The unpopularity of destocking is becoming inevitable as suggested by one of the Livestock Development Committee chairpersons that ‘we have to reduce cattle numbers as most grazing areas are now for human settlement’ (Chairperson, male, January 2018). Communal farmers view culling as a major setback. Failure to articulate negatives of not destocking and the subsequent benefits it brings deprives communal farmers of opportunities to earn income and invest in other pressing capitals. Lack of clarity on post-drought recovery strategies contributes to low uptake of destocking by communal cattle farmers. Farmers questioned why the AGRITEX department was emphasising on destocking without exploring other avenues that can save cattle. ‘Cattle defines a man’, (Farmer, female), insinuating that without cattle, one’s status and contribution to society is hardly noticed. Vetter (2013) concurs with findings that destocking is difficult to implement in communal areas and that resistance is likely to be experienced as communal farmers are affectionate with their livestock. Vetter and Bond (2012) share the same view that the majority of communal farmers have fewer cattle than they need; hence, it is imperative to note that while destocking uptake is a challenge because of the decline in grazing size, the value it brings has made it unavoidable. Cattle are a capital communal farmers can use to cushion themselves against persistent droughts and diversify livelihoods.

### Savings clubs

Savings clubs are not popular with communal cattle farmers as a strategy to counter drought threats despite strides by resilience building players to cultivate the culture through village savings and lending programmes. Communal farmers indicated that their income levels are too low to accommodate savings. Entrenched in communal farmers is mistrust with the banking sector following the erosion of savings in previous years especially 2008. Participants relived the 2008 period during which the majority of depositors’ resources were devalued, while some of the farmers’ investments were never recovered from banks. While ushering of the multi-currency era by the government in the year 2010 was welcomed, exorbitant bank charges discourage deposits and saving, hence farmers shy away. Of interest was that savings’ groups at their infancy were largely dominated by women of which the majority does not own cattle. The fear of keeping huge sums of money works against establishing savings clubs in communal spheres. Poor management of savings is another impediment to the expansion of the practice. In resonance, Lee (2011) professes that few savings clubs are healthily managed, while several appear to perform badly. Although savings clubs are perceived difficult to initiate, Hendricks and Chidiac (2011) aver that if the concept is embraced, it may enhance recognition of communal farmers by the formal financial sector as savers rather than borrowers which could escalates chances of being assisted. The practice has the propensity to inculcate a banking behaviour into the rural community and help farmers to diversify and invest in less vulnerable ventures. In addition, it offers a platform to drought exposed farmers to strengthen their financial management attributes. However, Hill, Hoddinott and Kumar (2013) argue that this practice is ineffective especially when the entire community is affected. The challenge with savings clubs is their propensity to attract mostly the better-off while excluding vulnerable cattle farmers whose buffer mechanisms are in most of the times compromised.

### Insurance

The adoption of insurance as a drought mitigation strategy is deemed luxurious and not a viable option. Further, discussions unravelled that cattle insurance is beyond the reach of most communal farmers as they had no guaranteed income to meet specified premiums. The high number of uninsured cattle against drought is not commensurate with risk levels posed by the hazard and the value communal farmers attach to livestock. There is limited understanding by communal farmers on the possible means of dealing with stressors and transference of drought risks. The restricted number of insurance companies offering livestock insurance services coupled with low-income levels within the communal farming community deters implementation of such mitigation practices. Hallegatte (2011) emphasises that insurance offers valuable means of hastening drought recovery other than relying on government assistance, as it spreads risks over time. Supportive institutions such as local government are encouraged to play a critical role of providing an enabling environment of dealing with any market and regulatory imperfections such as information asymmetries and low-level risk awareness amongst farmers. Such has the tendency to promote the uptake of insurance in communal areas.

### Fodder banks

Farmers acknowledged that the establishment of fodder banks was not a successful venture because of lack of knowledge and appropriate resources. On the contrary, interaction with extension officers revealed that theoretical programmes on fodder production were common though without translation into practice. In-depth analysis proved that stock owners with plots at a local irrigation scheme manage to grow fodder as the area is protected. The failure by the majority of communal farmers to plant fodder grass demonstrates lack of pro-activeness and fragility of coping mechanisms to the hazard even at lower thresholds. While communal farmers have expressed engaging varied drought coping mechanisms, they focus on unsustainable means that continuously expose and perpetuate vulnerability of cattle to drought shocks. Infrastructure to support this venture is close to non-existence as evidenced by high levels of uncontrolled grazing. In concurrence, one of the traditional leaders said: ‘dealing with animal intrusion is difficult’, (Tradisional leader, male, February 2018) implying deep rooted grazing management challenges. Grazing management constraints emanate from weak institutional arrangements which deepen poor organisation of farmers in utilising natural resources. Mbora and Lilleso (2007) support the view that communal farmers pay less attention to fodder banks and that their vulnerability to drought is, consequently, perpetuated. An example is Ethiopia where disinclination towards new approaches and technologies that seek to safeguard livestock against persistent drought is common (Dercon & Christiaensen 2011). This behaviour has triggered Peters et al. (2012) to challenge communal farmers to tap from the widely publicised livestock-related mitigation through improved pasture interventions. In light of this trend, extension agents’ contribution is always envisaged through establishing demonstration plots to encourage uptake of the fodder crops as a viable drought coping intervention.

### Credit facilities

Most communal farmers denied ever accessing credit lines to cushion livestock against drought. Despite the publicity and euphoria on Agri-bank loans targeting commercial and communal farmers, an insignificant number have benefitted as most communal livestock is not bankable because of the poor management practices, high incidences of disease, high mortality rates and failure to insure them. The limited access to credit facilities was induced by poor collateral which makes communal farming not attractive to banking institutions and other financiers. The land-holding system in communal areas places farmers at a disadvantage with no title to it, making it extremely difficult to attract credit lines from financial institutions. The thinking by communal farmers that cattle can be used as collateral does not hold given the vulnerability of the asset to drought and health shocks. Even successful farmers world over do require capital injection to make improvements on machinery to enhance production efficiency. Financial capital has become necessary even to communal farmers if they are to effectively cope with drought shocks. Private moneylenders deepen access to credit challenges by charging exorbitant costs to loans (Mago 2013). The impediment to accessing capital by communal farmers is compounded by lack of clearly laid down business plans and records to support their endeavour to run a commercial entity and attract financial capital. While local banks are encouraged to advance loans to communal farmers and devise means to use livestock as collateral, such a move has not been popular because of a plethora of reasons, which include poor disease control.

## Conclusion

Coping strategies implemented reveal limited investment in strengthening cattle management practices by communal farmers as they largely rely on indigenous ways which exacerbate the risks to drought. While farmers spoke highly and with passion about cattle, efforts to cushion livestock against drought shocks never matched the concern. There is high dependence on moisture-sensitive coping mechanisms such as dry land cropping and shared grazing which is a clear indication that the community is yet to embrace contemporary cattle rearing practices. The culture of safety is not inculcated in this community, given that savings clubs, insurance and establishment of fodder are not common drought amelioration practices. Coping mechanisms practiced reflect a poorly resourced and engaged farming community on modern cattle production and this has the propensity to relegate communal farmers to be mere spectators in the industry, despite holding a significant fraction of the national herd. Technical arms of government need to embrace different coping options as they lay a foundation for adaptation and inform their constituencies on best ways to integrate modern and traditional initiatives to enhance capacities to cope with drought. There is need to explore the influence of institutional frameworks in bolstering drought coping in communal cattle farming areas.
